# Risk of gastric and oesophageal adenocarcinoma following discontinuation of long-term proton-pump inhibitor therapy

**DOI:** 10.1007/s00535-022-01930-3

**Published:** 2022-10-18

**Authors:** Dag Holmberg, Fredrik Mattsson, Shaohua Xie, Eivind Ness-Jensen, Hashem El-Serag, Jesper Lagergren

**Affiliations:** 1 Department of Molecular Medicine and Surgery, Karolinska Institutet and Karolinska University Hospital, Retzius Street 13a, 17177 Stockholm, Sweden; 2 Department of Public Health and Nursing, NTNU, Norwegian University of Science and Technology, Levanger, Norway; 3 Department of Medicine, Levanger Hospital, Nord-Trøndelag Hospital Trust, Levanger, Norway; 4 Department of Medicine, Baylor College of Medicine, Houston, USA; 5 School of Cancer and Pharmacological Sciences, King’s College London, London, UK

**Keywords:** Proton-pump inhibitors, Oesophageal neoplasm, Gastric neoplasm, Prescription

## Abstract

**Background:**

There is uncertainty whether long-term use of proton-pump inhibitors can cause gastric adenocarcinoma (GAC) and oesophageal adenocarcinoma (OAC). This study aimed to determine how discontinuation of long-term PPI therapy influences the risk of GAC and OAC.

**Methods:**

This population-based cohort study included all long-term users of PPI therapy in Sweden in 2005–2018 was based on Swedish nationwide health registry data. The exposure was discontinuation of long-term PPI therapy, defined as no dispensation of PPI following inclusion and used as a time-varying variable, compared to remaining on PPI. Main outcomes were GAC and OAC, while oesophageal squamous cell carcinoma (OSCC) was included as a comparison outcome. Incidence rate ratios (IRR) with 95% CI adjusted for age, sex, comorbidity, obesity, diabetes, hyperlipidaemia, NSAIDs/aspirin, and statins were calculated with Poisson regression.

**Results:**

Among 730,176 long-term PPI users (mean age 65.6 years, 58.4% females) with 4,210,925 person-years at risk (median 5.5 person-years), 439,390 (60.2%) discontinued PPIs. In total, 495 developed GAC, 598 OAC, and 188 developed OSCC. PPI discontinuation was associated with decreased risk of GAC (IRR 0.81, 95% CI 0.67–0.98) and OAC (IRR 0.80, 95% CI 0.68–0.96), but not OSCC (IRR 1.10, 95% CI 0.82–1.49) compared to continued PPI use. Stratified analyses showed decreased point estimates across most age categories and both sexes for GAC and OAC risk among participants discontinuing PPI therapy.

**Conclusion:**

Discontinuation of long-term PPI therapy may decrease the risk of GAC and OAC, suggesting that physicians should consider ceasing prescribing long-term PPI in patients without continued indication for its use.

**Supplementary Information:**

The online version contains supplementary material available at 10.1007/s00535-022-01930-3.

## Introduction

Proton-pump inhibitors (PPIs) are among the most prescribed medications in the Western world [1, 2]. PPIs increase the pH of the otherwise acidic gastric juice by inhibiting the H^+^/K^+^ ATPase of the gastric parietal cells. The main indications for long-term PPI use are gastro-oesophageal reflux disease (GERD) and gastric ulcer prevention in long-term users of non-steroidal anti-inflammatory drugs (NSAIDs) or aspirin [3]. Long-term acid suppression by PPI therapy can lead to overproduction of the hormone gastrin and potentiation of the gastric carcinogen *Helicobacter pylori*, inducing fundic gland polyps and gastric atrophy. This might increase the risk of gastric adenocarcinoma, although results are contradictory [4]. Reflux of acidic gastric juice might cause erosive esophagitis and when the inflamed squamous cell epithelium remains exposed it can develop into columnar metaplasia, which might progress into dysplasia and ultimately oesophageal adenocarcinoma [5]. The limited literature on the relation between long-term PPI therapy and oesophageal adenocarcinoma has provided conflicting results, but recent larger studies have indicated an increased risk associated with long-term PPI therapy [6–8]. The other main histological type of oesophageal cancer, i.e., squamous cell carcinoma, has not been associated with PPI therapy.

Instead of examining PPI therapy as exposure, this study set out to assess the association between *discontinuation* of long-term PPI therapy and risk of gastric and oesophageal adenocarcinoma as compared to the risk in patients remaining on PPI therapy. The risk of oesophageal squamous cell carcinoma was examined for comparison reasons.

## Methods

### Design

This was a nationwide and population-based cohort study based on all healthcare in Sweden. Study participants were recruited between July 1, 2005 and December 31, 2016, and were followed up until December 31, 2018. The study was approved by the Ethical Review Board in Stockholm, Sweden. Informed consent was waived due to the registry-based design and the analysis of anonymized data.

### Data sources

The information came from four well-established Swedish national health data registries, maintained by the National Board of Health and Welfare. Data linkage between these registries was enabled by the unique personal identity number given to each Swedish resident upon birth or immigration.

- *The prescribed drug registry* provided data on dispensation of PPIs as well as NSAIDs, aspirin, and statins for this study. This registry records all prescribed medications in Sweden from its initiation in July 1, 2005 and onwards. The information is controlled by the Swedish eHealth Agency and the completeness and quality of the data are excellent [9].
- *The cancer registry* identified the three cancer outcomes in this study and all other cancer types (including those in the Charlson Comorbidity Index). This holds tumor-specific data and has 98% completeness for registration of gastric adenocarcinoma, oesophageal adenocarcinoma, and oesophageal squamous cell carcinoma [10, 11].
- *The patient registry* delivered data on diagnoses and surgical procedures included in this study. The data have been well validated for high completeness and accuracy [12].
- *The cause of death registry* provided data on mortality for censoring purposes. This registry records date of all deaths in Swedish residents with a completeness of 100% [13].

### Study cohort

The study cohort consisted of all individuals in Sweden using long-term PPI therapy during the study period according to the Prescribed Drug Registry. The definition of long-term PPI therapy was dispensation of ≥ 180 tablets of PPIs [*Anatomic Therapeutical Codes* (ATC) listed in online Appendix] in a single year. Only long-term users of PPI were included because short-term use is unlikely to be carcinogenic, and 180 tablets have been considered a reasonable cut-off for long-term use in previous publications [14, 15]. Cumulative use was counted from first dispensation of PPI and 1 year further. If dispensation of ≥ 180 tablets of PPI occurred in 1 year, that individual was included in the study cohort; if not, cumulative use was counted from the next PPI dispensation until 1 year further and so forth (Supplementary Figure). The source cohort was the Swedish Prescribed Drugs and Health Cohort (SPREDH), which includes all individuals with a prescription of PPI in Sweden [16]. Excluded were individuals aged < 40 years (because of the low incidence of gastric and oesophageal cancer in younger persons) and those with previous gastric or oesophageal cancer.

### Exposure

The study exposure was discontinuation of long-term PPI therapy, which was defined as the lack of dispensation of PPIs in the years following study inclusion. Participants dispensing any amount of PPI following inclusion were categorized as remaining on PPI therapy. PPI therapy was used as a time-dependent covariate and each individual study participants could therefore contribute person-time to both categories remaining on and discontinuing PPI therapy, which is recommended to avoid immortal time bias (Supplementary Figure) [17]. Person-time following a year without any dispensation of PPI was allocated to the PPI discontinuation category. If PPI was resumed in any following year, the subsequent person-time at risk was allocated to the group remaining on PPI therapy. The comparison group in all analyses were patients remaining on PPI therapy.

### Outcomes

Gastric adenocarcinoma, oesophageal adenocarcinoma and oesophageal squamous cell carcinoma were defined by a set of diagnosis codes according to the 7th version of the International Classification of Diseases (ICD-7), combined with histology codes signifying adenocarcinoma or squamous cell carcinoma (online Appendix). Person-time after the diagnosis date of any of the three outcome tumors was censored, meaning that each participant could contribute to only one outcome.

### Confounders

Twelve variables were considered potential confounders: age, sex, Charlson Comorbidity Index [18], tobacco smoking-related diagnoses, obesity, diabetes, hyperlipidaemia, Barrett’s esophagus, anti-reflux surgery, *Helicobacter pylori* eradication therapy, NSAIDs or aspirin use, and statin use. All ICD, ATC, and NOMESCO codes used to define the confounders are listed in the online Appendix.

### Statistical analysis

Poisson regression was used to calculate incidence rate ratios (IRR) with 95% confidence intervals (CI) as the measure of relative risk comparing discontinuation of long-term PPI therapy with continued long-term PPI use in relation to risk of the cancer outcomes. Follow-up started 1 year after inclusion into the study cohort and ended on the date of any of the three cancer outcomes, death, or end of study period, whichever occurred first. Tumors that developed during the first year following discontinuation or remaining on PPI therapy (i.e., upon cohort entry or resumption of PPI therapy) were censored to allow for latency (Supplementary Figure). The IRRs were adjusted for age (categorized in approximate quartiles), sex (male or female), comorbidity (Charlson comorbidity index 0, 1 or ≥ 2), obesity, hyperlipidaemia or diabetes (as a combined variable associated with obesity, yes and no), NSAID or aspirin use (yes or no), and statin use (yes or no). To assess effect modification, we conducted analyses stratified for age, sex and follow-up time. Because tobacco smoking-related diagnoses, Barrett’s esophagus, anti-reflux surgery, and *Helicobacter pylori* eradication therapy occurred in a too small frequency of study participants to be included in the regression model, participants exposed to these factors were instead excluded in sensitivity analyses. All variable definitions, categorizations and analyses adhered to a detailed study protocol finalized before initiation of the data management. The analyses were conducted by a senior biostatistician (FM) using the statistical package SAS version 9.4 (SAS Institute Inc., Cary, NC, USA).

## Results

### Participants

**Fig. 1:**
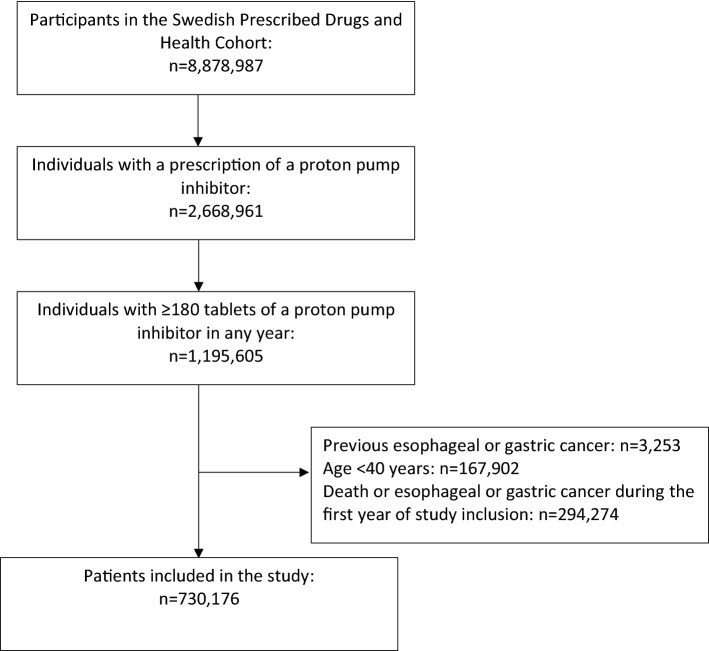
Selection of the study participants

The cohort included 730,176 long-term users of PPI (Fig. 1). These contributed 4,210,925 person-years at risk and the median follow-up was 5.5 years (IQR 2.7–9.0 years). The mean age at inclusion was 65.6 years and 58.4% were women (Table 1). Among the entire cohort, 439,390 (60.2%) participants discontinued long-term PPI therapy, and of these, 117,794 (26.8%) restarted PPI during follow-up. Long-term PPI therapy was discontinued in 8773 (2.0%) participants after anti-reflux surgery. Gastroduodenal ulcers developed in 15,015 (3.4%) participants who discontinued PPI, and in 26,070 (4.7%) participants remaining on PPI.

**Table 1:** Characteristics of individuals on discontinuing long-term proton-pump inhibitor (PPI) therapy compared to remaining on such therapy in Sweden

	Number (%)	
	Discontinuing PPI therapy	Remaining on PPI therapy
Total	439,390 (60.2)	558,869 (76.5)
Age (years)—mean ± SD	63.4 ± 12.7	66.3 ± 13.1
Sex		
Male	181,938 (41.4)	229,279 (41.0)
Female	257,452 (58.6)	329,590 (59.0)
Charlson comorbidity score		
0	317,078 (72.2)	368,414 (65.9)
I	84,800 (19.3)	120,392 (21.5)
≥ II	37,512 (8.5)	70,063 (12.6)
Tobacco smoking-related diagnosis	12,395 (2.8)	21,158 (3.8)
Obesity	10,004 (2.3)	12,967 (2.3)
Diabetes	56,693 (12.9)	86,677 (15.5)
Hyperlipidaemia	15,510 (3.5)	23,370 (4.2)
Barrett’s esophagus	157 (0.04)	321 (0.06)
Anti-reflux surgery	1,856 (0.4)	2,346 (0.4)
*Helicobacter pylori* eradication therapy	32,636 (7.4)	41,056 (7.4)
NSAID/aspirin use	194,308 (44.2)	268,388 (48.0)
Statin use	100,745 (22.9)	148,767 (26.6)
Follow-up (person-years)—mean ± SD	3.6 ± 2.8	4.7 ± 3.2
Gastric adenocarcinoma	157 (0.04)	338 (0.06)
Oesophageal adenocarcinoma	191 (0.04)	407 (0.07)
Oesophageal squamous cell carcinoma	72 (0.02)	116 (0.02)

*NSAID* non-steroidal anti-inflammatory drugs, *SD* standard deviation

### Risk of gastric adenocarcinoma

In total, 495 cases of gastric adenocarcinoma were identified, which corresponded to an incidence rate of 11.8 cases per 100,000 person-years. Compared with patients remaining on long-term PPI therapy, discontinuation of long-term PPI therapy was associated with a decreased risk of gastric adenocarcinoma (adjusted IRR 0.81, 95% CI 0.67–0.98) (Table 2). Apart from PPI discontinuation, younger age, female sex, and NSAID/aspirin use were associated with decreased risk of gastric adenocarcinoma in this cohort of long-term users of PPI (Table 3).

**Table 2:** Incidence rate ratio (IRR) and 95% confidence interval (CI) of gastric adenocarcinoma among individuals discontinuing proton-pump inhibitors compared to individuals remaining on proton-pump inhibitors (reference group)

	Discontinuing PPI therapy			Remaining on PPI therapy			Incidence rate ratio (95% CI)^a^
	Person-years at risk	Events (*n*)	Incidence rate (per 100,000 person-years)	Person-years at risk	Events (*n*)	Incidence rate (per 100,000 person-years)	
Overall	1,602,711	157	8.00	2,608,353	338	12.96	0.81 (0.67–0.98)
Age (in years)							
≤ 56	548,664	16	2.92	699,145	33	4.72	0.60 (0.33–1.10)
57–66	450,361	39	8.60	764,494	71	9.29	0.92 (0.62–1.36)
67–76	358,662	61	17.01	660,068	125	18.94	0.88 (0.65–1.20)
≥ 77	245,024	41	16.73	484,645	109	22.49	0.74 (0.52–1.06)
Sex							
Male	674,576	87	12.90	1,065,170	195	18.31	0.76 (0.59–0.98)
Female	928,135	70	7.54	1,543,183	143	9.27	0.88 (0.66–1.18)
Follow-up (in years)							
1–3	472,353	37	7.83	858,515	100	11.65	0.76 (0.52–1.10)
3–5	419,317	43	10.25	640,655	85	13.27	0.85 (0.59–1.23)
> 5	711,040	77	10.83	1,109,183	153	13.79	0.82 (0.62–1.08)

^a^Adjusted for age, sex, Charlson comorbidity index, obesity, diabetes, hyperlipidaemia, NSAID/aspirin use and statin use

**Table 3:** Risk factors associated with gastric and oesophageal cancer among long-term users of proton-pump inhibitor (PPI) therapy

	Adjusted incidence rate ratio (95% CI)^a^		
	Gastric adenocarcinoma (*n* = 495)	Oesophageal adenocarcinoma (*n* = 598)	Oesophageal squamous cell carcinoma (*n* = 188)
Discontinuation of PPI therapy			
No	1.00 (reference)	1.00 (reference)	1.00 (reference)
Yes	0.81 (0.67–0.98)	0.80 (0.68–0.96)	1.10 (0.82–1.48)
Age			
≤ 56	1.00 (reference)	1.00 (reference)	1.00 (reference)
57–66	2.32 (1.65–3.25)	2.46 (1.88–3.21)	2.80 (1.72–4.56)
67–76	4.76 (3.46–6.56)	2.99 (2.28–3.92)	2.95 (1.79–4.85)
≥ 77	5.63 (4.04–7.84)	3.11 (2.33–4.16)	2.55 (1.49–4.36)
Sex			
Male	1.00 (reference)	1.00 (reference)	1.00 (reference)
Female	0.50 (0.42–0.60)	0.20 (0.16–0.24)	0.54 (0.41–0.73)
Charlson comorbidity score			
0	1.00 (reference)	1.00 (reference)	1.00 (reference)
I	0.98 (0.78–1.22)	1.32 (1.08–1.61)	1.78 (1.25–2.53)
≥ II	1.24 (0.94–1.64)	1.64 (1.28–2.10)	3.13 (2.07–4.73)
Obesity/diabetes/hyperlipidaemia			
No	1.00 (reference)	1.00 (reference)	1.00 (reference)
Yes	1.25 (0.97–1.61)	1.24 (1.0–1.55)	0.97 (0.65–1.45)
NSAID/aspirin use			
No	1.00 (reference)	1.00 (reference)	1.00 (reference)
Yes	0.76 (0.63–0.92)	0.78 (0.66–0.93)	1.10 (0.81–1.48)
Statin use			
No	1.00 (reference)	1.00 (reference)	1.00 (reference)
Yes	0.90 (0.73–1.11)	0.90 (0.74–1.09)	0.74 (0.52–1.05)

^a^Adjusted for age, sex, Charlson comorbidity index, obesity, diabetes, hyperlipidaemia, NSAID/aspirin use and statin use

### Risk of oesophageal adenocarcinoma

A total of 598 participants developed oesophageal adenocarcinoma, which corresponded to an incidence rate of 14.2 cases per 100,000 person-years. Discontinuation of long-term PPI use was associated with a decreased risk of oesophageal adenocarcinoma compared to continuation of PPI therapy (adjusted IRR 0.80, 95% CI 0.68–0.96) (Table 4). In this cohort, younger age, female sex, and NSAID/aspirin use were also associated with a decreased risk of oesophageal adenocarcinoma, while increased Charlson Comorbidity Index, obesity, diabetes, and hyperlipidaemia were associated with an increased risk (Table 3).

**Table 4:** Incidence rate ratio (IRR) and 95% confidence interval (CI) of oesophageal adenocarcinoma among individuals discontinuing proton-pump inhibitors compared to individuals remaining on proton-pump inhibitors (reference group)

	Discontinuing PPI therapy			Remaining on PPI therapy			Incidence rate ratio (95% CI)^a^
	Person-years at risk	Events (*n*)	Incidence rate (per 100,000 person-years)	Person-years at risk	Events (*n*)	Incidence rate (per 100,000 person-years)	
Overall	1,602,711	191	11.92	2,608,353	407	15.60	0.80 (0.68–0.96)
Age (in years)							
≤ 56	548,664	33	6.01	699,145	44	6.29	0.94 (0.60–1.48)
57–66	450,361	63	16.7	764,494	128	16.7	0.83 (0.61–1.12)
67–76	358,662	49	13.7	660,068	147	22.3	0.60 (0.43–0.83)
≥ 77	245,024	46	18.8	484,645	88	18.2	1.03 (0.72–1.47)
Sex							
Male	674,576	162	24.0	1,065,170	305	28.6	0.90 (0.74–1.09)
Female	928,135	29	3.12	1,543,183	102	6.61	0.51 (0.34–0.77)
Follow-up (in years)							
1–3	472,353	34	7.20	858,515	105	11.65	0.63 (0.43–0.92)
3–5	419,317	48	11.45	640,655	86	13.27	0.92 (0.65–1.31)
> 5	711,040	109	15.33	1,109,183	216	19.47	0.81 (0.64–1.02)

^a^Adjusted for age, sex, Charlson comorbidity index, obesity, diabetes, hyperlipidaemia, NSAID/aspirin use and statin use

### Risk of oesophageal squamous cell carcinoma

During follow-up, 188 cases of oesophageal squamous cell carcinomas were detected, resulting in an incidence rate of 4.5 cases per 100,000 person-years. Discontinuation of long-term PPI therapy did not decrease the risk (adjusted IRR 1.10, 95% CI 0.82–1.48), and no clear trend was observed in the stratified analyses (Table 5).

**Table 5:** Incidence rate ratio (IRR) and 95% confidence interval (CI) of oesophageal squamous cell carcinoma among individuals discontinuing proton-pump inhibitors compared to individuals remaining on proton-pump inhibitors (reference group)

	Discontinuing PPI therapy			Remaining on PPI therapy			Incidence rate ratio (95% CI)^a^
	Person-years at risk	Events (*n*)	Incidence rate (per 100,000 person-years)	Person-years at risk	Events (*n*)	Incidence rate (per 100,000 person-years)	
Overall	1,602,711	72	4.49	2,608,353	116	4.45	1.10 (0.82–1.48)
Age (in years)							
≤ 56	548,664	5	0.91	699,145	17	2.43	0.38 (0.14–1.04)
57–66	450,361	32	7.11	764,494	32	4.19	1.73 (1.06–2.83)
67–76	358,662	21	5.86	660,068	40	6.06	0.97 (0.57–1.65)
≥ 77	245,024	14	5.71	484,645	27	5.57	1.06 (0.56–2.02)
Sex							
Male	674,576	43	6.37	1,065,170	64	6.01	1.17 (0.79–1.72)
Female	928,135	29	3.12	1,543,183	52	3.37	1.02 (0.65–1.60)
Follow-up (in years)							
1–3	472,353	16	3.39	858,515	33	3.84	1.00 (0.55–1.83)
3–5	419,317	22	5.25	640,655	29	4.53	1.30 (0.75–2.27)
> 5	711,040	34	4.78	1,109,183	54	4.87	1.04 (0.67–1.59)

^a^Adjusted for age, sex, Charlson comorbidity index, obesity, diabetes, hyperlipidaemia, NSAID/aspirin use and statin use

### Sensitivity analyses

The sensitivity analyses excluded 81,881 participants with tobacco smoking-related disorders, Barrett’s esophagus, anti-reflux surgery, or *Helicobacter pylori* eradication therapy, leaving 648,295 participants followed for median 5.8 (IQR 2.8–9.3) person-years. The adjusted IRRs after discontinuing long-term PPI were 1.05 (95% CI 0.80–1.38) for gastric adenocarcinoma, 0.83 (95% CI 0.66–1.04) for oesophageal adenocarcinoma, and 0.93 (95% CI 0.60–1.44) for oesophageal squamous cell carcinoma.

## Discussion

This first study on the topic indicates that discontinuing long-term PPI therapy is associated with a decreased risk of gastric adenocarcinoma and oesophageal adenocarcinoma, and not of oesophageal squamous cell carcinoma.

Strengths of the study include the population-based design and large sample size, where virtually all long-term users of prescribed and dispensed PPIs in Sweden were included, which counteracted selection bias and improved statistical power. Defining drug use as dispensations from a registry rather than by questionnaire had the advantages of prospective data collection, thus avoiding misclassification due to issues of recalling, and actual dispensing (and paying for) the prescribed medication, which reduced non-compliance. The use of nationwide complete registries ensured long and complete follow-up for all endpoints. Including oesophageal squamous cell carcinoma as a control outcome with no association with PPI use facilitated the interpretation, and the lack of association between discontinuation of long-term PPI use and this cancer supported the validity of the findings for gastric and oesophageal adenocarcinoma.

Using PPI as a time-varying covariate and the use of 1 year latency minimized the risk of immortal time and latency bias [17]. Causality can rarely be claimed in observational studies examining the effect of PPI on oesophageal and gastric adenocarcinoma, but several measures were attempted to avoid bias from confounding and other sources. First, only long-term users of PPI were included in the cohort, thus avoiding short-term users. Second, contrasting studies assessing the influence of PPI use on the risk of developing these tumors, the results were adjusted for NSAID, aspirin, and statin therapy. This could otherwise be a major concern because these medications are often prescribed alongside long-term PPI therapy and may decrease the risks of these tumors, which was the case of NSAID/aspirin in this study [19, 20]. Third, the adjustment for the key confounding variables age, sex, Charlson Comorbidity Index, obesity, diabetes and hyperlipidaemia, tobacco smoking, Barrett’s esophagus, anti-reflux surgery, or *Helicobacter pylori* eradication therapy ensured that virtually all known confounders were accounted for.

Other weaknesses of the study include the inability to accurately examine duration and cumulative dosage of PPI use, given the methodological pitfalls and the risk of introducing immortal time using this approach in cohort studies [21]. The inability to assess over-the-counter prescriptions of PPIs should not be a major problem in this study, because PPIs are available over-the-counter only at low doses (< 1 DDD), small packages (maximum 28 tablets), and at a significantly higher cost compared to prescribed PPIs in Sweden. Thus, the vast majority of patients on long-term PPI therapy have prescriptions. Nevertheless, it is not possible to exclude that some participants classified as discontinuers in fact were on small amounts of PPI bought without prescription. We also acknowledge that a supposed higher volume of endoscopies among patients remaining on PPI may lead to earlier detection of oesophageal and gastric cancer, which could lead to a slight overestimation of the incidence among these patients compared to those who stopped using PPI.

The decreased risk of gastric adenocarcinoma following discontinuation of long-term PPI use was hypothesized and also observed in the main analysis. Among the exclusion variables, the main risk factor for gastric adenocarcinoma is *Helicobacter pylori* infection, while smoking is a weak risk factor, and Barrett’s esophagus and anti-reflux surgery are not associated with this tumor. *Helicobacter pylori* or its treatment is not an indication for long-term PPI use and should therefore not be a major confounder for the association between PPI discontinuation and risk of gastric adenocarcinoma, although some patients with gastroduodenal ulcers may be prescribed PPI indefinitely. Nevertheless, after excluding participants with *Helicobacter pylori* eradication therapy and tobacco smoking-related disorders in a sensitivity analysis, the association was attenuated to null for gastric adenocarcinoma. Although this sensitivity analysis had low power, the association should be interpreted with some caution.

Several previous studies have indicated a strong association between PPI use and gastric adenocarcinoma. In this study, we found an incidence of gastric adenocarcinoma in patients remaining PPI in parity with the incidence in the Swedish general population (of which approximately 6% use PPI), but the risk was slightly lower in patients discontinuing PPI [22]. A meta-analysis of observational studies found that long-term PPI therapy was associated with a 43% increased risk of gastric cancer (odds ratio 1.43, 95% CI 1.23–1.66) [4]. Two more recent and large cohort studies have provided further evidence of an increased risk of gastric cancer among long-term users of PPIs. A cohort study from Hong Kong among 63,397 *Helicobacter pylori*-eradicated participants, found that long-term use of PPIs was associated with an over twofold increased risk of gastric cancer (hazard ratio 2.44, 95% CI 1.42–4.20) [23]. A Swedish cohort study identified a threefold increased risk of gastric cancer among 797,067 individuals on maintenance PPI therapy compared to the corresponding general population (standardized incidence ratio 3.38, 95% CI 3.23–3.53), and the association remained in participants who used PPIs for indications without any association with gastric cancer, e.g., GERD (standardized incidence ratio 3.04, 95% CI 2.80–3.31) [24]. The lower effect sizes observed in the present study compared to studies assessing the association between PPI use and gastric and oesophageal adenocarcinoma may indicate better control of methodological issues such as confounding and time-related biases in this study.

Some mechanisms can explain a potentially decreased risk of gastric adenocarcinoma after discontinuation of long-term PPI therapy. First, discontinuation can normalize the elevated gastrin levels associated with long-term PPI use [25, 26], which in turn can counteract parietal cell hyperplasia and polyposis [27, 28]. Second, atrophic gastritis caused by *Helicobacter pylori* can progress in PPI users and promote carcinogenesis, which could be counteracted by PPI discontinuation [29–31]. This mechanism is supported by the lack of association between PPI discontinuation and gastric adenocarcinoma when participants with a history of *Helicobacter pylori* were excluded in the present study. While *Helicobacter pylori* eradication therapy decreases the risk of gastric adenocarcinoma, it should be noted that atrophic gastritis represents a late stage and may still progress to intestinal metaplasia following eradication therapy [32, 33].

Similar to the results of gastric adenocarcinoma, the risk of oesophageal adenocarcinoma was decreased in this study. However, the study population represented a group at clearly higher risk of oesophageal adenocarcinoma compared to the corresponding Swedish general population [22], likely due to a higher prevalence of gastroesophageal reflux disease. While the incidence was higher in patients remaining on PPI, both women and men discounting PPI retained an increased risk of oesophageal adenocarcinoma compared to the general Swedish population. Decreased point estimates in the sensitivity analysis and almost all subgroups of participants further support the finding that the risk of oesophageal adenocarcinoma is decreased after stopping long-term PPI therapy. The literature examining the role of PPI use in the etiology of oesophageal adenocarcinoma has provided conflicting results. Three large observational studies have suggested increased risks of oesophageal adenocarcinoma among long-term PPI users but the results have been met with skepticism because of the risk of confounding by indication [6, 8, 34]. In patients with Barrett’s esophagus, a randomized clinical trial (AspECT) with low statistical power found no difference in risk of oesophageal adenocarcinoma comparing high-dose with low-dose PPI [35], and a meta-analysis indicated a decreased risk of oesophageal adenocarcinoma among PPI users [36]. A biological mechanism that can explain the finding of a risk reduction of oesophageal adenocarcinoma after discontinuation of PPI therapy is normalization of the elevated gastrin levels associated with long-term PPI use [25, 26]. Gastrin upregulates oesophageal expression of the pro-inflammatory enzyme COX-2, which is considered a promotor of oesophageal adenocarcinoma development. Thus, normalized gastrin levels may counteract COX-2 activity and decrease oesophageal adenocarcinoma risk, explaining why PPI discontinuation could prevent this tumor [37–39].

Other substances present in the refluxate among patients with GERD, particularly bile acids, have been proposed as causative of oesophageal adenocarcinoma. Biliary reflux may be a cause of refractory GERD not responding well to PPI therapy, and most patients with GERD also have pathologically increased biliary reflux [40]. These acids may contribute to oesophageal adenocarcinoma development by inducing oesophageal squamous cells to differentiate to intestinal-like cells and promoting inflammation through activation of nuclear factor kappa light-chain-enhancer of activated B cells (NF-κB) [40, 41]. Animal models have demonstrated that oesophageal exposure to reflux of bile acids results in Barrett’s esophagus and subsequent adenocarcinoma [42, 43]. Gastric juices have been shown to be even dose-dependently protective against oesophageal adenocarcinoma in rats with biliary reflux [43]. Consequently, some of the increased risk of oesophageal adenocarcinoma in patients continuing PPI may be attributed to increased toxicity of bile acids in the absence of gastric acid.

The possible risk reduction of oesophageal and gastric adenocarcinoma in individuals discontinuing long-term PPI observed in this study are not indiscriminately generalized to all patients on long-term PPI therapy. PPIs are well tolerated by most patients and usually offer effective symptom relief for GERD and is essential in the prevention of upper gastrointestinal bleeding among patients on anticoagulants. In these settings, the benefits of long-term PPI will outweigh any increase in relative risk of gastric and oesophageal adenocarcinoma. Nevertheless, this study may still have clinical implications for many individuals. Long-term PPI therapy is exceedingly common and has further increased in recent years, and is often used for off-label indications [2]. The findings from this study should encourage physicians to consider stopping long-term PPI therapy in the absence of a clear indication for its use. Yet, given that this is the first study directly assessing how discontinuation of PPI use influences cancer risk, the findings need to be confirmed in future research.

In conclusion, this large population-based cohort study indicates that discontinuation of long-term PPI therapy is associated with a decreased risk of both gastric adenocarcinoma and oesophageal adenocarcinoma, although residual confounding cannot be excluded. In the absence of a clear indication for long-term PPI therapy, the need for PPI therapy should be reconsidered to avoid unnecessary cases of gastric and oesophageal adenocarcinoma.

## Supplementary Information

Below is the link to the electronic supplementary material.

Supplementary file 1 (PDF 232 kb)

## Data Availability

The data that support the findings of this study are available from The Swedish Board of Health and Welfare. Restrictions apply to the availability of these data, which were used under license for this study. Data are available from the authors with the permission of The Swedish Board of Health and Welfare.
