# Supplementary material for: Risk of gastric and oesophageal adenocarcinoma following discontinuation of long-term proton-pump inhibitor therapy
Source: J Gastroenterol. 2022 Oct 18;57(12):942–51. doi: 10.1007/s00535-022-01930-3 (PMC9663349; doi:10.1007/s00535-022-01930-3)
Supplement: Supplementary file 1 — (PDF 232 kb) [file 535_2022_1930_MOESM1_ESM.pdf]

Appendix

1. Drugs included in the study

**Proton-pump inhibitors**		
**Drug name**	**ATC code**	**Defined daily dose**
Omeprazole	A02BC01	20 mg
Pantoprazole	A02BC02	40 mg
Lansoprazole	A02BC03	30 mg
Rabeprazole	A02BC04	20 mg
Esomeprazole	A02BC05	30 mg

**Anti-inflammatory medication**		
**Drug name**	**ATC code**	**Defined daily dose**
Non-steroidal anti-inflammatory drugs	M01A	N/A
Acetylsalicylic acid	B01AC06	1 tablet (independent of strength)

2. ICD-codes included in the study

**Charlson comorbidity index**	
**Disease**	**ICD-10 codes**
Myocardial infarction	I21-I22, I12, I252
Heart failure	I11, I13, I255, I43, I50, I517
Peripheral vascular disease	I70-I73, I770, I771, K558, K559, Z958, Z959, K551, R02
Cerebrovascular disease	G45-G46, I60-I69
Dementia	F00-F03, G30-G31, A810, F051
Chronic obstructive pulmonary disease	I26-I27, J40-J47, J60-J67, J684, J701, J703
Rheumatic disease	M05-M06, M32-M36, M09, M120, M315
Liver disease	K70-K71, B18, I85, I864, I982, K721, K29, K76, R162, Z944
Diabetes (type 1 and 2)	E10-E14
Hemi/paraplegia	G81-G83, G114
Renal disease	N01, N03, N05, N07-N08, N171, N172, N18, N19, N25, Z49, Z940, Z992
AIDS/HIV	B20-B24
Malignancy	C00-C26, C30-C34, C37-C41, C43, C45-C58, C60-C76, C80-C85, C88, C90-C97
Metastatic tumor	C77-C79

Gastric and esophageal cancer		
**Disease**	**ICD-7 codes**	**WHO/HS/CANC/C24.1 histology code**
Esophageal adenocarcinoma	150, 1511	096
Esophageal squamous cell carcinoma	150	146
Gastric adenocarcinoma	1510, 1518, 1519	096

Cohort entry

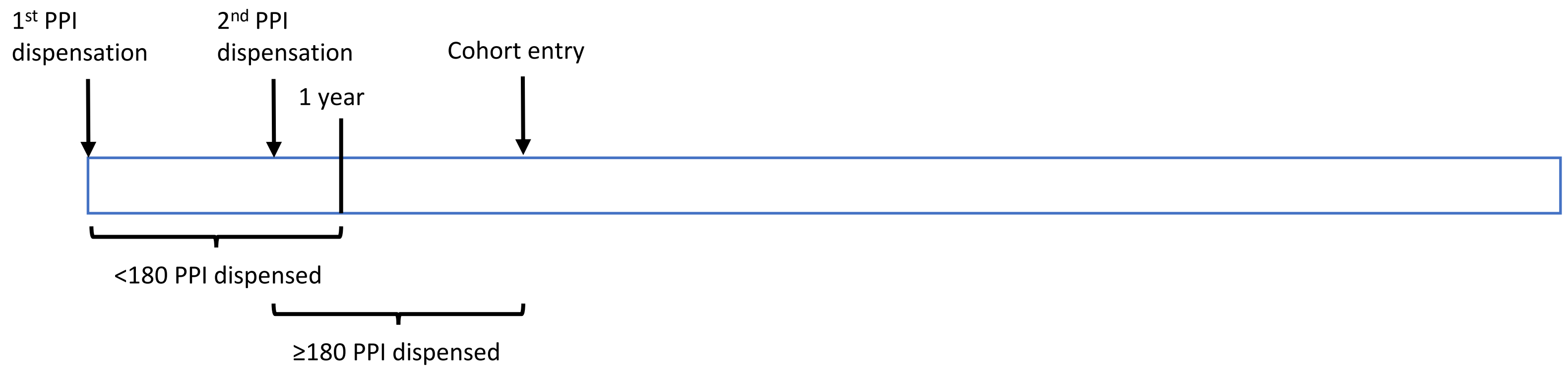

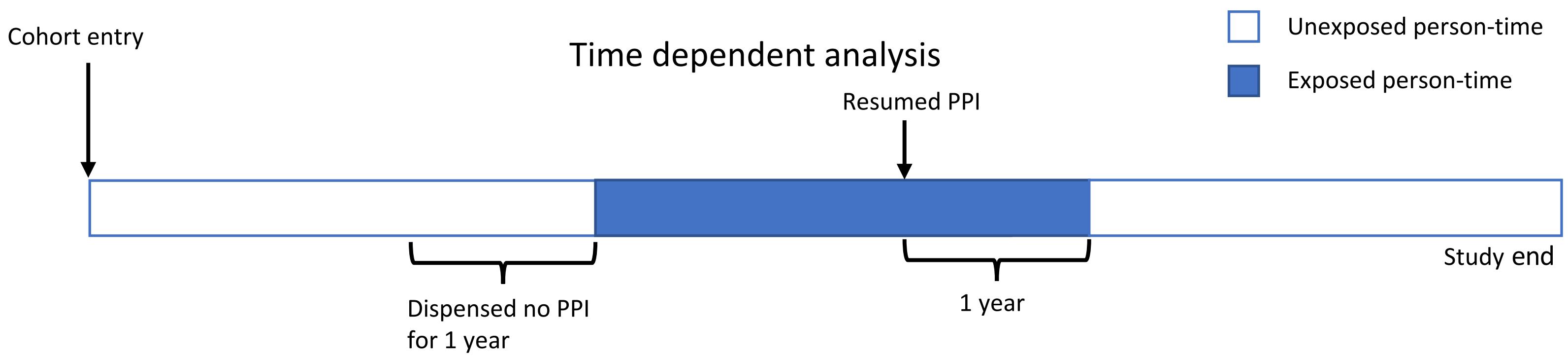
Cohort entry
Time dependent analysis
Unexposed person-time
Exposed person-time
Resumed PPI
Study end
Dispensed no PPI for 1 year
1 year

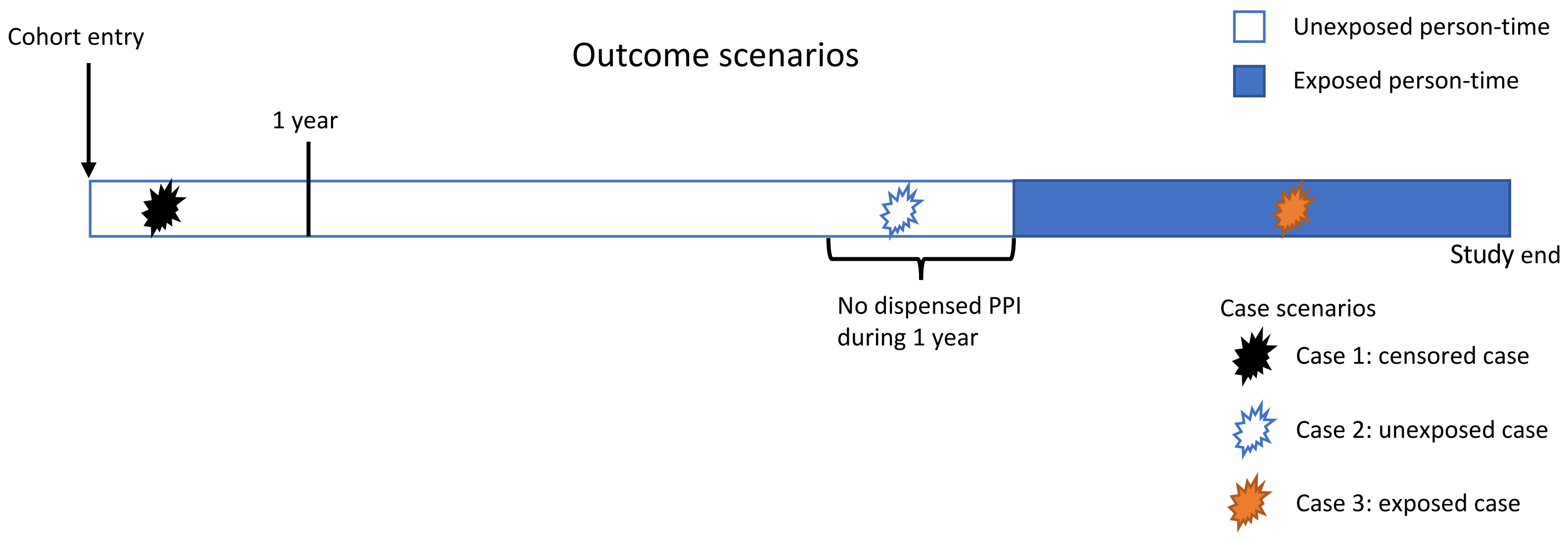
Cohort entry
Outcome scenarios
Unexposed person-time
Exposed person-time
1 year
Study end
No dispensed PPI during 1 year
Case scenarios
Case 1: censored case
Case 2: unexposed case
Case 3: exposed case
